# Genomic mutation features identify distinct BRCA-associated mutation characteristics in endometrioid carcinoma and endometrioid ovarian carcinoma

**DOI:** 10.18632/aging.203710

**Published:** 2021-11-27

**Authors:** Canhui Cao, Ruidi Yu, Wenjian Gong, Dan Liu, Xiaoxue Zhang, Yong Fang, Yu Xia, Wei Zhang, Qinglei Gao

**Affiliations:** 1 Cancer Biology Research Center, Key Laboratory of the Ministry of Education, Tongji Hospital, Tongji Medical College, Huazhong University of Science and Technology, Wuhan, Hubei, China; 2 Department of Gynecology and Obstetrics, Tongji Hospital, Tongji Medical College, Huazhong University of Science and Technology, Wuhan, Hubei, China

**Keywords:** endometrioid carcinoma, endometrioid ovarian carcinoma, genome mutation, BRCA, immune response

## Abstract

Although endometrioid carcinoma (EC) and endometrioid ovarian carcinoma (EnOC) display similar pathological features, their molecular characteristics remain to be determined. Somatic mutation data from 2777 EC, 423 EnOC, and 57 endometriosis patients from the Catalogue of Somatic Mutations in Cancer (COSMIC) dataset were analyzed and showed similar profiles with different mutation frequencies among them. By using 275 overlapping mutated genes, EC was clustered into two groups with different disease outcomes and different clinical characteristics. Although BRCA-associated mutation characteristics were identified in both EC and EnOC, the mutation frequencies of BRCA1 (P=0.0146), BRCA2 (P=0.0321), ATR (P=3.25E-11), RAD51 (P=3.95E-08), RAD1 (P=0.0003), TP53 (P=6.11E-33), and BRIP1 (P=2.90E-09) were higher in EnOC. Further analysis showed that EnOC cell lines with BRCA-associated mutation characteristics were more sensitive to poly ADP-ribose polymerase (PARP) inhibitors than EC cell lines, including olaparib, talazoparib, rucaparib, and veliparib. Moreover, based on BRCA-associated mutational and transcriptomic profiles, EC with BRCA-associated mutational burdens shows lower levels of immune cell infiltration, higher expression of immunosuppressive checkpoint molecules and worse prognosis than EC without BRCA mutation. Our study comprehensively analyzed the genome mutation features of EC and EnOC and provide insights into the molecular characteristics of EC and EnOC.

## INTRODUCTION

Endometrial cancer is a common malignancy in the female genital organs [1]. It is a group of epithelial malignant tumors that occur in the endometrium, also known as uterine body cancer. Perimenopausal and postmenopausal women are the high-risk populations [2]. Endometrioid carcinoma (EC) is the most common histological subtype, accounting for 80% of endometrial cancer. It usually has an adenoidal or villous tubular structure, accompanied by crowded and complicated branch structures [3]. Endometrioid ovarian carcinoma (EnOC) which has similar pathologic features to EC, is a rare ovarian cancer subtype that accounts for only 10% of epithelial ovarian cancer [4, 5].

Despite the similar pathology, the molecular characteristics of EC and EnOC remain to be determined. Endometrial carcinoma has high mutation frequencies of the ARID1A, CTNNB1, KRAS, PTEN, and PIK3CA genes [6]. In addition to these genes, EnOC is also altered frequently at SOX8 [7]. TP53 rarely mutated in EC, but frequently mutated in EnOC [6, 7]. Selected exon capture sequencing found that compared with low-grade EnOC, PTEN mutations were more frequent in low-grade EC, while CTNNB1 mutations showed the opposite trend [8]. However, in terms of genome-wide molecular characteristics and mutation profiles, the relationship between EC and EnOC needs to be further investigated.

BRCA1/2 are tumor suppressor genes involved in DNA damage repair. High-risk mutations that disable an important error-free DNA repair process (homology-directed repair), significantly increase the risk of cancer [9]. Additionally, BRCA1/2 mutations are related to increased susceptibility to endometrial and ovarian carcinoma [10, 11]. Polyadenine diphosphate ribose polymerase (PARP) inhibitors can block single-strand repair [12, 13], thereby inducing synthetic lethality in BRCA1/2 deficient tumor cells [14, 15]. In addition, BRCA mutation carriers were more sensitive to platinum-based chemotherapy [16]. For EC and EnOC, the difference and connection of BRCA status, including the prognostic difference caused by mutation status, have not been clarified.

In this research, we conducted genome variation, functional enrichment, gene ontology (GO) pathway enrichment, Kaplan–Meier plotter, coexpression, interaction, and tumor immunology analyses of the accessible database Cancer Somatic Mutation Catalog (COSMIC), to explore the mutation profiles of the two morphologically similar tumor types, EC and EnOC.

## RESULTS

### Genomic mutation features in EC, EnOC, and En

**Figure 1:**
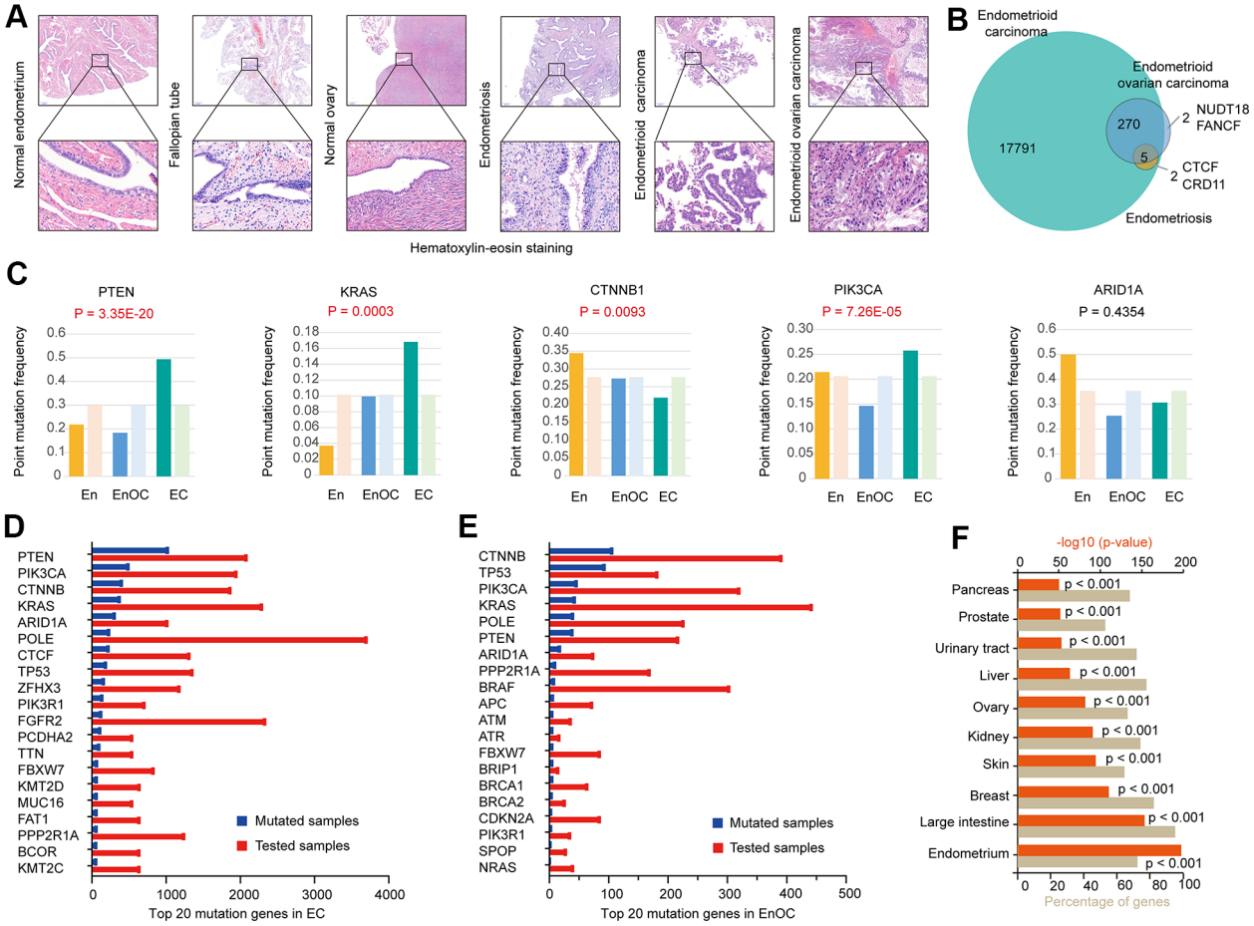
**Genomic mutation features in EC, EnOC, and En.** (**A**) Hematoxylin-Eosin staining of EC and EnOC and En samples. (**B**) Mutant genes of EC and EnOC and En. (**C**) Point mutation frequency of five common mutant genes (PTEN, KRAS, CTNNB1, PI3K3CA, and ARID1A) in EC, EnOC, and En, the left column was the observed point mutation frequency, and the right was the expected point mutation frequency. (**D**) Top 20 mutation genes in EC, which were rank ordered following the number of mutated samples. (**E**) Top 20 mutation genes in EnOC. (**F**) The mutation percentage of the 17791 genes in various organs.

The pathology of endometrioid carcinoma was similar to that of endometrioid ovarian carcinoma (Figure 1A). We identified 17791 mutant genes in EC (Supplementary Table 1), including 275 overlapping mutant genes with EnOC (99.28%, Supplementary Table 2) and 7 overlapping with En (100.00%, Supplementary Table 3). Five (71.43%) overlapping mutant genes were identified in EC, En and EnOC. Two unique mutant genes, NUDT18 and FANCF, only occurred in EnOC. In addition, CTCF and CRD11 mutations were only found in En and EC (Figure 1B). PTEN, KRAS, CTNNB1, PIK3CA, and ARID1A were common mutated genes in EC, EnOC, and En. Compared with EnOC and En, EC had a higher point mutation frequency in PTEN (P = 3.35E-20), KRAS (P = 0.0003), and PIK3CA (P = 7.26E-05), and lower frequency in CTNNB1 (P = 0.0093, Figure 1C). The top 20 most frequent mutant genes differed between EC and EnOC (Figure 1D, 1E). We imported these 17791 mutant genes into FunRich (v3.1.3) for the enrichment analysis. These 17791 mutant genes most often appeared in the endometrium (p<0.001), followed by the breast (p<0.001), ovary (p<0.001), urinary tract (p<0.001), and other organs (p<0.001, Figure 1F).

### Gene enrichment analysis of 275 common mutated genes and their association with EC prognosis

**Figure 2:**
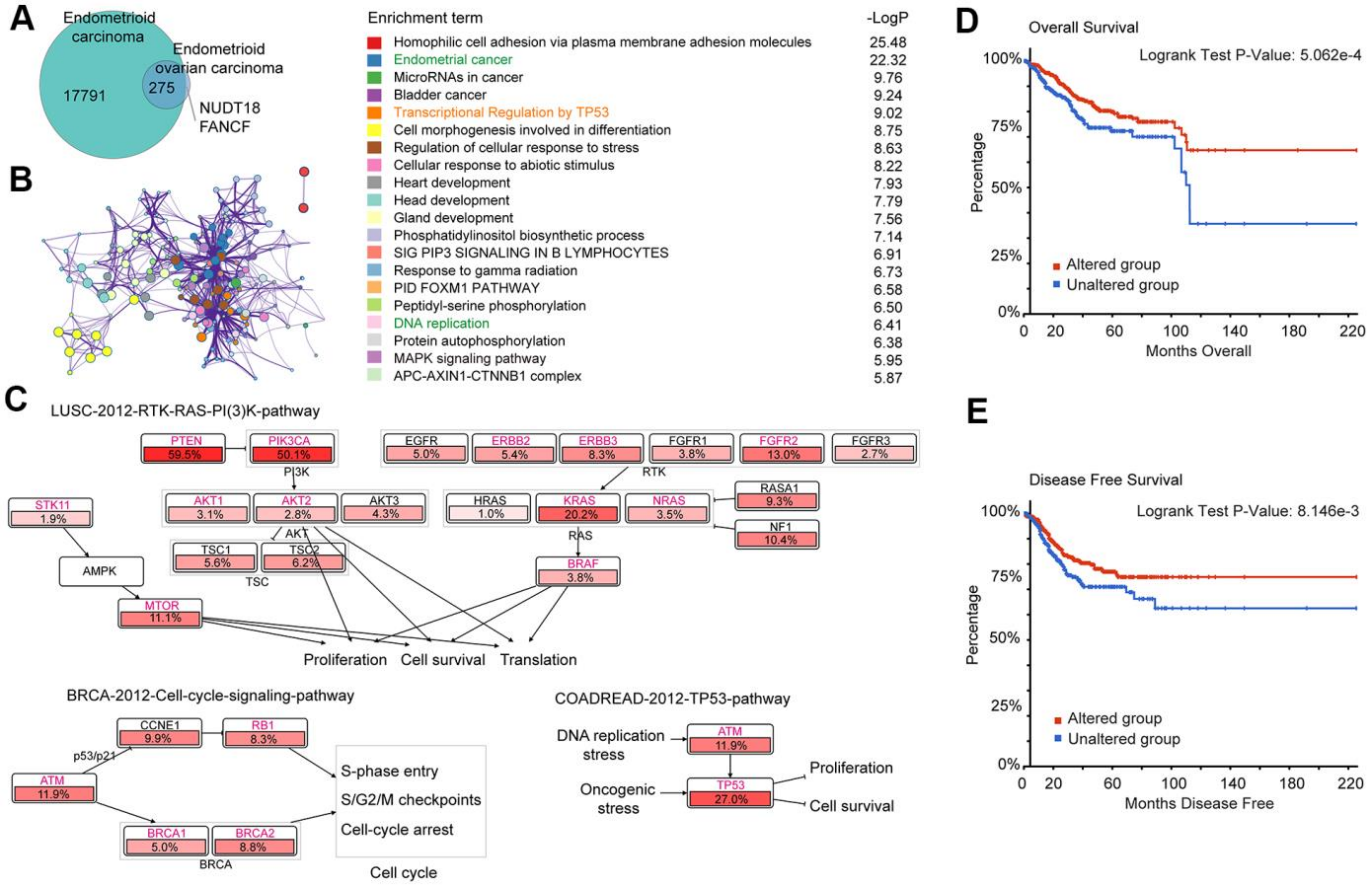
**Gene enrichment analysis of 275 common mutation genes and their association with prognosis in EC.** (**A**) Mutant genes of EC and EnOC. (**B**) Protein-protein interaction (PPI) network and enriched terms of the 275 overlapped mutant genes of EC and EnOC. (**C**) Pathway enrichment analysis of the 275 genes in EC. The genes marked in red belonged to the above-mentioned 275 genes, and the percentages indicated the proportion of mutations in this gene in EC. (**D**, **E**) Overall survival and disease-free survival analysis of the altered and unaltered group. Patients with these 275 gene mutations were classified as the altered group, and those without were classified as the unaltered group.

A total of 275 genes were identified as common mutant genes between EC and EnOC (Figure 2A). To further explore the biological functions and signaling pathways of the 275 common mutant genes, Gene Ontology and Kyoto Encyclopedia of Genes and Genomes (KEGG) pathway enrichment analyses were performed. These genes were enriched in endometrial cancer (p=3.311E-26), transcriptional regulation by TP53 (p=9.550E-10), DNA replication (p=3.890E-7), and other functions (Figure 2B). The 275 common mutant genes (Figure 2A) were significantly enriched in the following pathways in endometrial carcinoma: (1) LUSC-2012-RTK-RAS-PI(3)K-pathway, which regulates the functions of proliferation, in which the mutation frequency of PTEN was 59.5% and PIK3A was 50.1%; (2) BRCA-2012-cell-cycle-signaling-pathway, which regulates the cell cycle on S-phase entry, S/G2, M checkpoints, and cell-cycle arrest, in which the mutation frequency of ATM was 11.9% (highest frequency in this pathway); (3) COADREAD-2012-TP53-pathway, which plays a role in cell proliferation and survival, under DNA replication stress, in which the mutation frequency of TP53 was 27.0% (Figure 2C). In addition, the 275 common mutated genes were also enriched in the BRCA-2012-RTK-RAS-PI(3)K-pathway which regulates JNK/JUN mediated apoptosis, proliferation, and apoptosis evasion (Supplementary Figure 1A), and the COADREAD-2012-WNT-signaling-pathway which regulates the proliferation and the stem/progenitor phenotype (Supplementary Figure 1B). For these 275 genes, the overall survival (OS, p=5.062E-4, Figure 2D) and disease-free survival (DFS, p=8.146E-3, Figure 2E) of the patients with alterations were better than those without alterations. In addition, in EC, there were significant differences between the altered group and unaltered group in various aspects, such as cancer type and stage, histologic type and grade, and new neoplasm event post initial therapy (Supplementary Figure 2 and Supplementary Table 4).

### Distinct BRCA-associated mutation characteristics were identified in EC and EnOC

**Figure 3:**
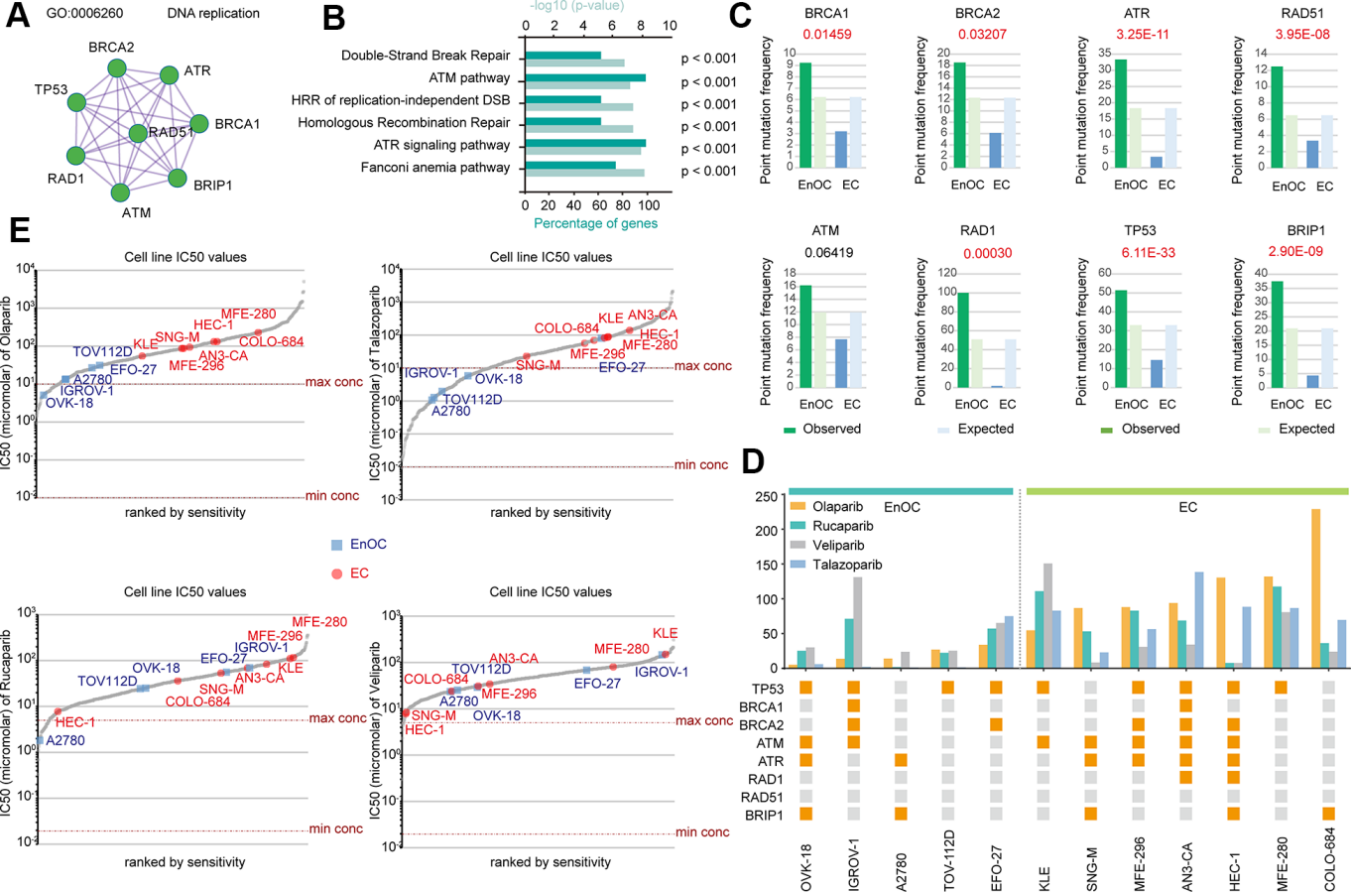
**Distinct BRCA-associated mutation characteristics and PARPi sensitivity in EC and EnOC.** (**A**) The PPI network of eight BRCA-related genes. (**B**) GO enrichment analysis of the eight genes. (**C**) Point mutation frequency of these genes in EC and EnOC. (**D**) The mutant status of these eight genes and IC50 values in different cell lines derived from EC and EnOC. Orange square: with a mutation; gray square: without a mutation. (**E**) The IC50 values of various cell line to Olaparib, Talazoparib, Rucaparib and veliparib. Cell lines derived from EnOC were marked in blue and cell lines derived from EC were marked in red.

There were 275 common mutant genes related to DNA damage repair functions (Figure 2B). Among those genes, BRCA1, BRCA2, RAD1, RAD5, ATM, ATR, BRIP1, and TP53 could form a protein-protein interaction network related to DNA replication (Figure 3A). The above eight genes associated with DNA damage repair were associated with double-strand break repair (p < 0.001), the ATM pathway (p < 0.001), homologous recombination of replication-independent double strand break (p < 0.001), homologous recombination repair (p < 0.001), the ATR signaling pathway (p < 0.001), and the Fanconi anemia pathway (p < 0.001, Figure 3B). The point mutation frequencies of BRCA1 (P = 0.0146), BRCA2 (P = 0.0321), ATR (P = 3.25E-11), RAD51 (P = 3.95E-08), RAD1 (P = 0.0003), TP53 (P = 6.11E-33), and BRIP1 (P = 2.90E-09) were much higher in EnOC than in EC (Figure 3C). The mutant statuses of cell lines derived from EnOC (OVK-18, IGROV-1, A2780, TOV112D, and EFO-27) and EC (KLE, SNG-M, MFE-296, AN3-CA, HEC-1, MFE-280, and COLO-684) were not the same (Figure 3D) with cell lines from EnOC more sensitive to PARPis, especially olaparib and talazoparib (Figure 3D, 3E).

### BRCA-associated features identified two signatures with different mutation patterns and disease outcomes in EC

**Figure 4:**
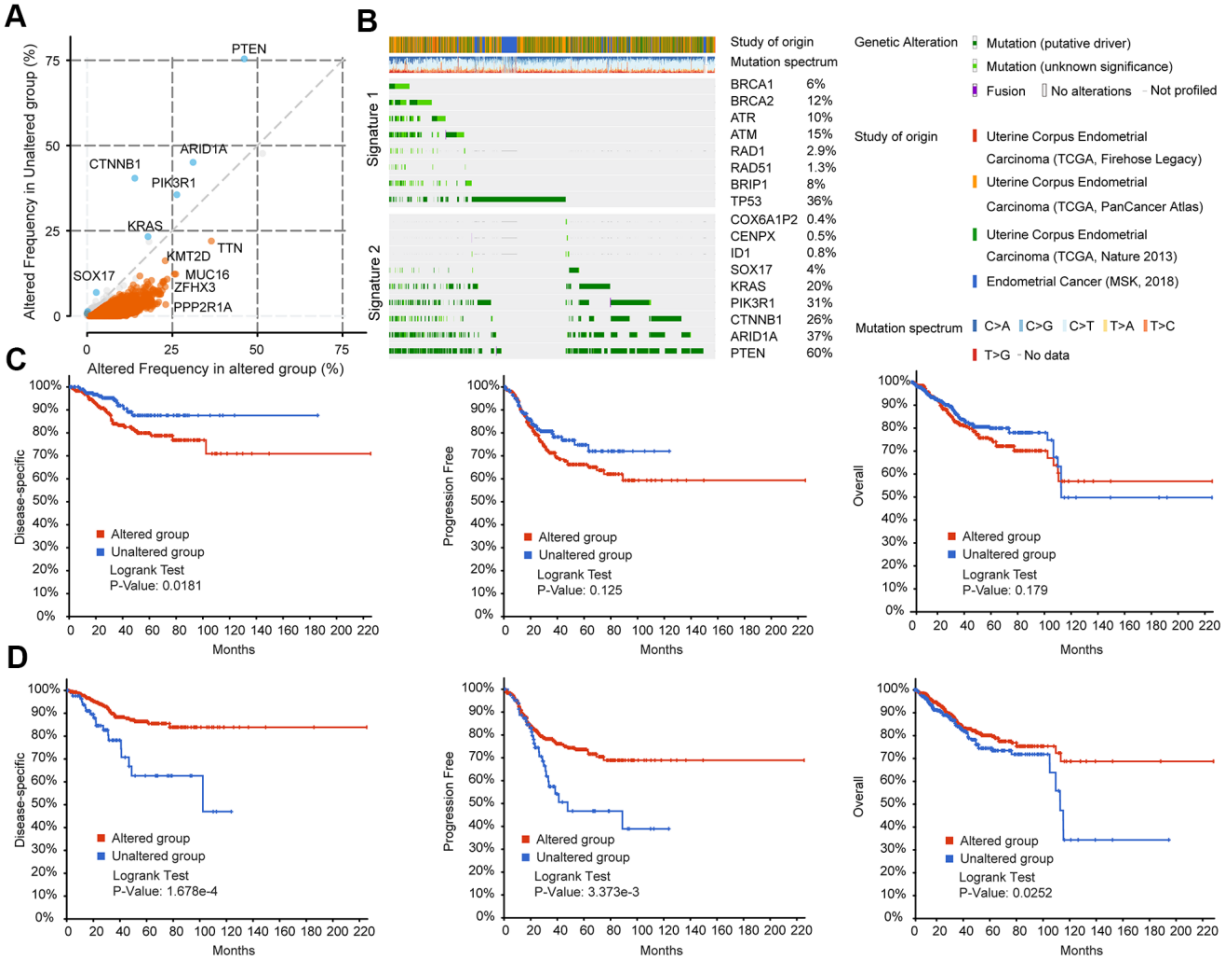
**BRCA-associated features identified two signatures with different mutation patterns and disease outcomes in EC.** (**A**) Co-expression analysis of the altered group and unaltered group in EC. Altered group: patients with mutations in the above BRCA-related genes; unaltered group: patients without mutations in the above BRCA-related genes (**B**) Oncoprint visual summary of Signature 1 and Signature 2 in EC patients. (**C**) DFS, PFS, and OS analysis in EC patients with or without Signature 1 alterations. (**D**) DFS, PFS, and OS analysis in EC patients with or without Signature 2 alterations.

Mutations in TIN, KMT2D, MUC16, ZFHX3, PPP2RIA, and other genes frequently occurred simultaneously with mutations in BRCA-associated genes (BRCA1, BRCA2, RAD1, RAD5, ATM, ATR, BRIP1, and TP53) (Figure 4A). In contrast, COX6A1P2, GENPX, 1D1, SOX17, KRAS, PIK3R1, CTNNB1, AR1D1A, and PTEN mutations rarely occurred simultaneously with mutations in the eight BRCA-associated genes (Figure 4A). According to the mutation status of the eight BRCA-associated genes, EC could be clustered into two groups. The mutation spectrum of the 9 non co-occurrent mutated genes is partially opposite to the mutation spectrum of BRCA-related genes, and these 9 genes could also divide EC patients into two groups with different mutational characteristics. Therefore, we clustered the eight BRCA-associated genes into Signature 1 and clustered the nine non co-occurring mutant genes into Signature 2 (Figure 4B). Supplementary Figure 3A shows the details of genetic alterations and the mutation spectrum of the five cooccurring mutant genes (TTN, KMT2D, ZFHX3, MUC16, and PPP2R1A) in EC (Supplementary Figure 3A). With these two signatures, EC patients could be divided into different subgroups. Patients with alterations in the Signature 1 gene had poorer disease-specific survival (DSS, p=0.0181), progression-free survival (PFS, p=0.125), and OS (p=0.179) than those without alterations (Figure 4C). In contrast, the DSS, PFS, and OS of patients with alterations in the genes of Signature 2 were much better than those of patients without alterations (Figure 4D). Clustered by the mutation status of the five cooccurring mutant genes, there were no significant differences between the altered and unaltered groups (Supplementary Figure 3B).

### BRCA-associated features identified two signatures with different immune-associated expression patterns in EC

**Figure 5:**
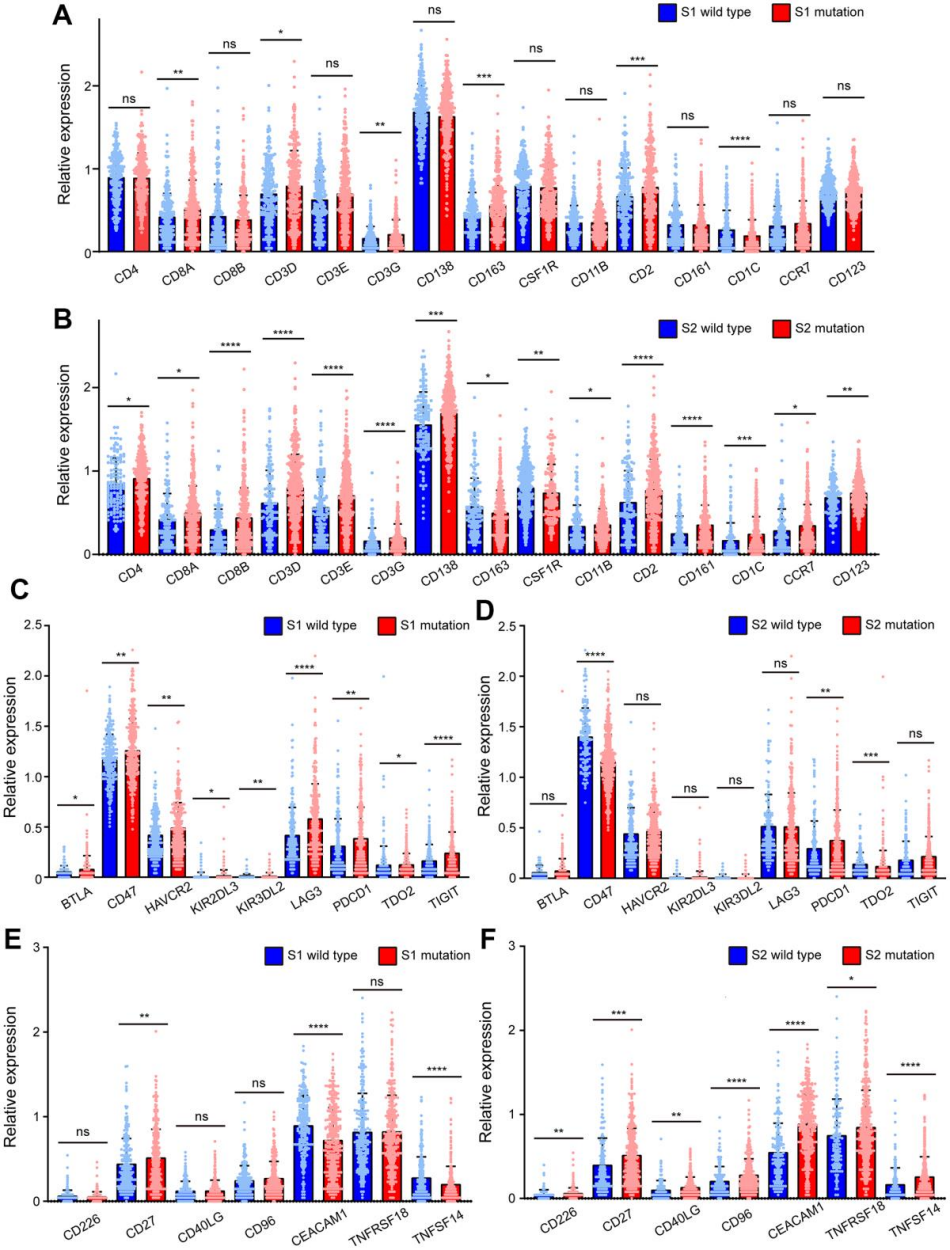
**BRCA-associated features identified two signatures with different immune-associated expression patterns in EC.** (**A**) The expression of CD4, CD8A, CD8B, CD3D, CD3E, CD3G, CD138, CD163, CSF1R, CD11B, CD2, CD161, CD1C, CCR7, CD123 of EC patients clustered by Signature 1. (**B**) The expression of CD4, CD8A, CD8B, CD3D, CD3E, CD3G, CD138, CD163, CSF1R, CD11B, CD2, CD161, CD1C, CCR7, CD123 of EC patients clustered by Signature 2. (**C**) The expression of BTLA, CD47, HAVCR2, KIR3DL3, KIR3DL2, LAG3, PDCD1, TDO2, TIGIT of EC patients clustered by Signature 1. (**D**) The expression of BTLA, CD47, HAVCR2, KIR3DL3, KIR3DL2, LAG3, PDCD1, TDO2, TIGIT of EC patients clustered by Signature 2. (**E**) The expression of CD226, CD27, CD40LG, CD96, CEACAM1, TNFRSF18, TNFSF14 of EC patients clustered by Signature 1. (**F**) The expression of CD226, CD27, CD40LG, CD96, CEACAM1, TNFRSF18, TNFSF14 of EC patients clustered by Signature 2. * P<0.05; ** P<0.01; ***P<0.001; ****P<0.0001; ns indicated that there was no significant statistical difference.

To investigate the causes of the two different prognostic trends of S1 and S2, surface molecules were used to reflect the level of immune cell infiltration, including T lymphocytes, B lymphocytes, natural killer cells, dendritic cells, and macrophage markers. There were no significant differences in immune cell infiltration between patients with or without S1 mutations except for CD8A, CD3D, CD3G CD163, CD2, and CD1C (Figure 5A). CD163 is a marker of M2-like macrophages, which are related to immune escape and tumor progression. Patients with S1 mutations had higher CD163 expression. CD1C is a surface marker of dendritic cells, and its expression is lower in patients with S1 mutations. CD8A, CD3D, and CD3G are surface markers of T lymphocytes, and CD2 is a marker of NK cells. Patients with the S2 mutation showed significantly higher immune cell infiltration levels including T lymphocytes, B lymphocytes, natural killer cells, dendritic cells, and macrophages, than patients without the S2 mutation (Figure 5B). This finding consistent with the better prognosis in patients with S2 mutations.

Immune checkpoint molecules were also used to investigate the causes of the two different prognostic trends of S1 and S2. The expression of immunosuppressive checkpoint molecules, such as BTLA, CD47, HAVCR2, KIR3DL3, KIR3DL2, LAG3, PDCD1, TDO2, and TIGIT, in patients with S1 mutations was much higher than that in S1 wild-type patients (Figure 5C). However, significant differences were not observed between patients with or without S2 mutations except for CD47, PDCD1, and TDO2. CD47 and TDO2 were lower in patients with S2 mutations than in those without mutations, which is inconsistent with the trend of S1 mutations (Figure 5D). However, in patients with S1 mutations, the levels of these molecules were not significantly different, except for CD27, CEACAM1, and TNFSF14. CEACAM1 and TNFSF14 were lower in patients with S1 mutations than in those with wild type S1, which is inconsistent with the trends of S2 (Figure 5E). It is worth noting that in tumors with the S2 mutation, the levels of immune checkpoint molecules for immune activation, including CD226, CD27, CD40LG, CD96, CEACAM1, TNFRSF18, and TNFSF14, were significantly higher than those in tumors without the S2 mutation (Figure 5F).

Tumors with mutations at TTN, MUC16, ZFHX3, and PPP2R1A, which occurred simultaneously with Signature 1, had CD8^+^T cell infiltration levels similar to those without mutations (Supplementary Figure 4A). It is worth noting that the infiltration of CD8^+^ T cells in tumors with TP53 mutations was significantly less than that in tumors without TP53 mutations (p <0.0001). The infiltration of macrophages (p <0.0001) and dendritic cells (p <0.001) showed a similar infiltration trend (Supplementary Figures 4A, 5B). The mutation count (p=4.99E-7), aneuploidy score (p=5.55E-8), and MSI sensor score (p=1.481E-6) of tumors with alterations in the Signature 1 gene were all higher than those of tumors without alterations (Supplementary Figure 4C). Meanwhile, compared with the unaltered group clustered by Signature 1, there were more copy-number variants, amplifications, and deletions in the altered group (Supplementary Figure 4D). The infiltration levels of CD8^+^ T cells in patients with mutations at PTEN (p <0.05), ARID1A (p <0.05), CTNNB1 (p <0.0001), and PIK3R1 (p <0.05), which belong to Signature 2, were higher than those without mutations (Supplementary Figure 4E). The cumulative survival of EC patients with a CD8^+^ T cell infiltration rate of more than 50% was significantly better than that of patients with less CD8^+^ T cell infiltration (p = 0.022, Supplementary Figure 4F). Additionally, to some extent, mutations at TP53 (gene in S1), PTEN, ARID1A, CTNNB1, PIK3R1, KRAS (genes in S2), TTN, MUC16, ZFHX3 and PPP2R1A were related (to some extent) to the infiltration of B cells, CD8^+^ T cells, CD4^+^ T cells, macrophages, neutrophils, or dendritic cells (Supplementary Figure 5A–5J).

### Evaluation of the effectiveness of olaparib in EC and EnOC *in vitro*

**Figure 6:**
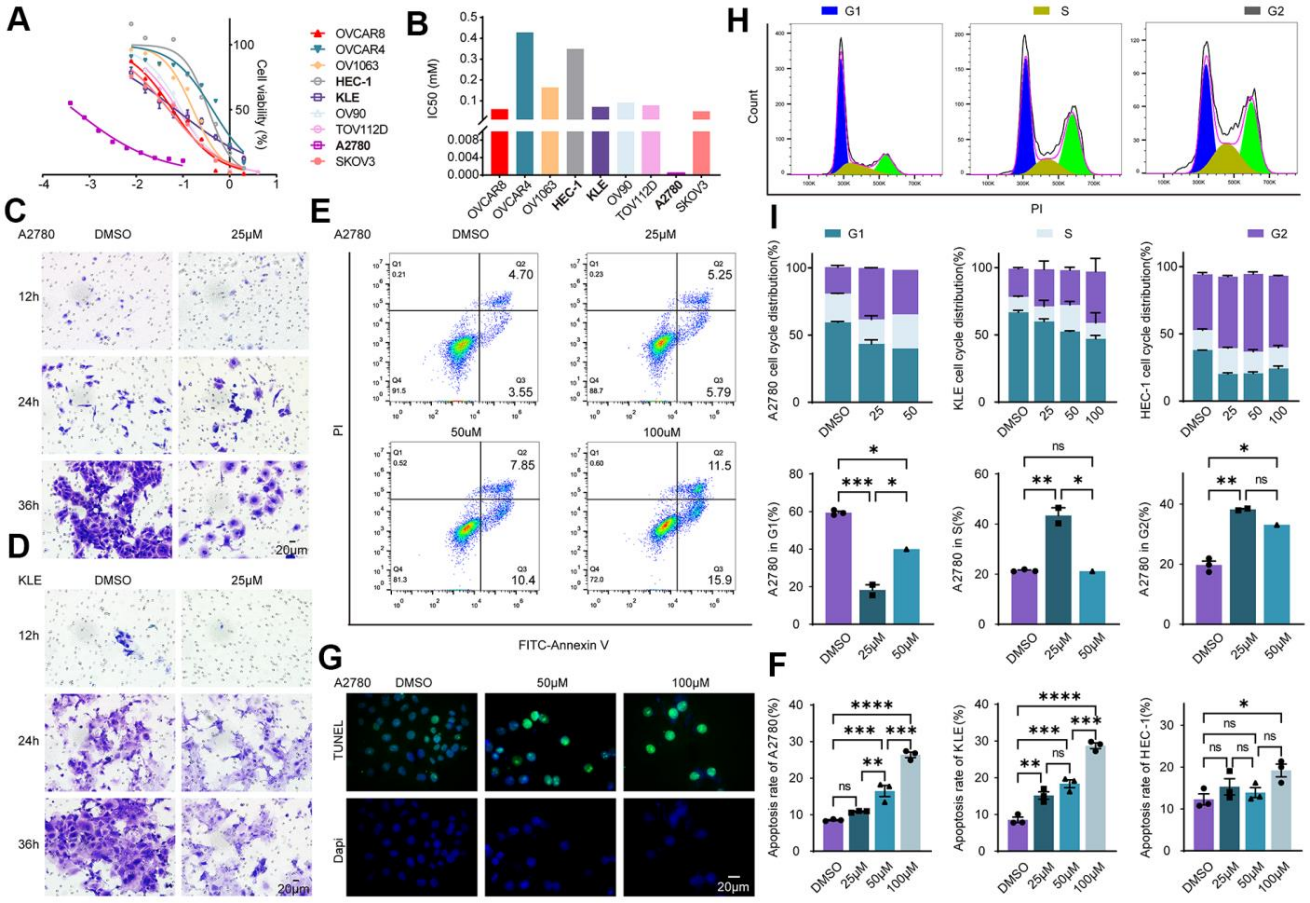
**Evaluation of the effectiveness of olaparib in EC and EnOC *in vitro*.** (**A**) Cell viability curve. (**B**) The IC50 value of cell lines to Olaparib. (**C**, **D**) Cell migration evaluated with Transwell assay in A2780 and KLE. (**E**, **F**) Apoptosis assay with flow cytometry and the statistical chart. (**G**) Apoptosis analysis with TUNEL assay. (**H**, **I**) Cell cycle analysis and the statistical chart. * P<0.05; ** P<0.01; ***P<0.001; ****P<0.0001; ns indicated that there was no significant statistical difference.

The mutant statuses of cell lines derived from EnOC and EC were not the same (Figure 3D). Cell lines from EnOC were more sensitive to PARPis, especially olaparib and talazoparib (Figure 3D, 3E). *In vitro* experiments were performed in the A2780 a cell line derived from EnOC [17], KLE and HEC-1 cell lines derived from EC, and other ovarian cancer cell lines (OVCAR8, OVCAR4, OV1063, OV90, TOV112D, SKOV3). A2780 cells were more sensitive to Olaparib than KLE and HEC-1 cells and had the smallest IC50 value among the above cell lines, which was consistent with the results from the database (Figure 6A, 6B). In A2780 and KLE cells, the number of cells that underwent migration was lower in the olaparib-treated group than in the DMSO-treated group (Figure 6C, 6D). In addition, flow cytometry showed that the apoptosis rates (both early and late apoptosis) of A2780, KLE and HEC-1 were higher in the Olaparib-treated group than in the DMSO group (Figure 6E, 6F, and Supplementary Figure 6A, 6B). We also verified these findings using a TUNEL assay, and similar results were found (Figure 6G, and Supplementary Figure 6C–6E). After treatment with olaparib, the percentages of cells in the S and G2 phases increased, and the percentage of cells in the G1 phase decreased in A2780, KLE and HEC-1 cells (Figure 6H, 6I, and Supplementary Figure 7A–7C).

### Olaparib promotes DNA damage in EC and EnOC and inhibits tumor growth *in vivo*

**Figure 7:**
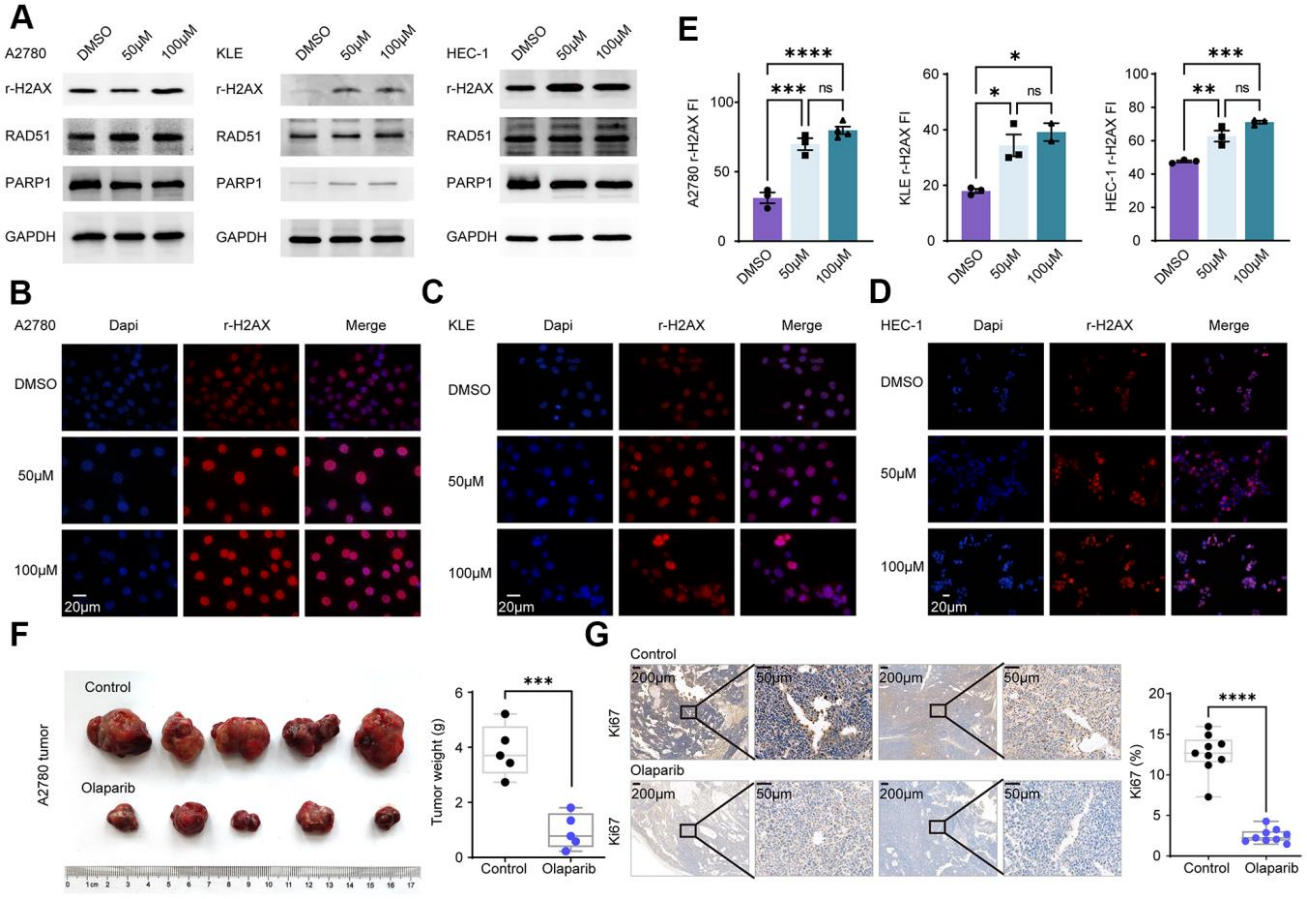
**Olaparib promotes DNA damage in EC and EnOC and inhibits tumor growth *in vivo*.** (**A**) Western blot of cell lines treated with DMSO and Olaparib. (**B**–**E**) Immunofluorescence and quantification of r-H2AX in A2780, KLE and HEC-1. (**F**) Tumor volume and weight after treated with Olaparib and Vehicle. (**G**) Immunohistochemistry analysis of Ki67 and the statistical chart. * P<0.05; ** P<0.01; ***P<0.001; ****P<0.0001; ns indicated that there was no significant statistical difference.

Olaparib, a PARP inhibitor mainly acts on the DNA damage repair pathway. Cells were separately treated with DMSO, 50uM olaparib and 100uM olaparib, followed by Western blot assay. The expression of r-H2AX and RAD51 was increased in EC and EnOC cell lines after the olaparib treatment (Figure 7A). Cellular immunofluorescence assays were also performed in A2780, KLE and HEC-1 cells, and the fluorescence intensity (FI) of r-H2AX was elevated after the olaparib treatment (Figure 7B–7E).

A2780 cells were injected subcutaneously into nude mice and the mice, which were divided into two groups: the control group and the olaparib group. The olaparib treatment resulted in a decrease in tumor volume and tumor weight. The tumors were then harvested and tested based on an immunohistochemistry analysis. Tumor Ki67 expression was significantly decreased in the olaparib-treated group compared to the control group (Figure 7F, 7G).

## DISCUSSION

Our research comprehensively compared the genome mutation features of EC and EnOC, which had mutations in BRCA1/2-associated genes. Based on the BRCA status, EC could be categorized into groups with different mutation patterns and clinical outcomes and was associated with different levels of immune cell infiltration, expression profiles of immune checkpoint molecules and sensitivity to PARPis. These findings might provide a reference for molecular targeted therapy and immunotherapy for EC and EnOC.

The pathological features of EC and EnOC were highly comparable. However, the genome-wide molecular characteristics and mutation profiles of EC and EnOC were not clearly articulated. Regarding the molecular characteristics, EC and EnOC presented some similarities, with up to 275 common mutant genes related to DNA damage repair [4, 5]. However, the mutation profiles of EC and EnOC were not the same. The point mutation frequencies of PTEN, KRAS, and PIK3CA in EC were higher than those in EnOC. which was consistent with previous study that PTEN and CTNNB1 mutations were significantly different in low-grade ovarian endometrioid carcinomas and low-grade endometrial endometrioid carcinomas [8]. Mutations in PTEN occurred in 59% of EC, which was lower than previous findings (69-80%) [6, 18]. However, the mutation frequencies of EC at KRAS, CYNB1, PIK3CA, and ARID1A were similar to those in previous studies [18].

The 275 common mutant genes were enriched in pathways regulating proliferation, cell cycle on S-phase entry, S/G2, M checkpoints, evading apoptosis, and stem/progenitor phenotype. The RAS-PI(3)K pathway was the most frequently mutated pathway in EC in our research. It was reported that molecular aberrations in the RAS-PI(3)K pathway existed in 80-95% of ECs [19]. The enriched pathways play a pivotal role in the occurrence and development of endometrial cancer, thus representing attractive and promising therapeutic targets, such as PARP inhibitors, and mTOR inhibitors [7, 20].

The BRCA status of EC and EnOC was not the same. The mutation frequencies of these eight BRCA associated genes in EnOC were higher than those in EC, and EnOC cell lines were more sensitive to PARPis. This finding indicated that EnOC patients with higher mutation frequencies of BRCA-related genes might benefit more from PARP inhibitors than EC patients. Consistent with the SOL1 Phase III clinical trial advanced ovarian cancer patients with BRCA1/2 mutations who experience remission after initial platinum-containing treatment could benefit from olaparib [21]. Some promising results were also found in the efficiency of PARPis in EC, with frequent mutations at PTEN and TP53 [22]. According to the NCCN guidelines, PARPis is recommended for patients with stage II, III and IV EnOC post primary treatment and patients with platinum-sensitive relapses. However, the use of PARP inhibitors in EC was not reflected. Therefore, our findings may provide additional reference data for the application of PARPis in EC and EnOC.

In the era of precision medicine, PARPis in combination with immune checkpoint inhibitors, have revolutionized the outcome of cancers including EC [22, 23]. The levels of immunosuppressive checkpoint molecules were lower in patients with BRCA-associated gene mutations, which was related to poorer prognosis in EC. In contrast, patients with S2 mutations expressed higher immune checkpoint molecules for immune activation, which was associated with a better prognosis. In addition, emerging evidence has demonstrated that elevated tumor infiltrating lymphocytes could rescue the prognosis in cancer [24]. DNA damage response deficiency also acts as a stimulator of interferon genes, which increase the immune infiltrate [22, 25]. The levels of immune cell infiltrations were lower in patients with BRCA-associated gene mutations, accompanied by poorer prognosis in EC. In contrast, patients with S2 mutations had higher immune cell infiltration levels and a better prognosis than patients without S2 mutations. Therefore, our results provide a reference for the combination of PARPi with immune checkpoint inhibitors in EC.

Despite the positive aspects, there were some limitations in our research. First, our research was a descriptive study and we did not demonstrate why different mutation patterns led to different prognoses or how the mutation patterns of BRCA-related genes affected tumor immunity and PARP inhibitor sensitivities. In addition, the relationship between S1 or S2 mutation and immune response was not verified.

In conclusion, this study comprehensively analyzed the genome mutations of EC, EnOC, and En and identified 275 common mutant genes between EC and EnOC. The mutation frequencies of these mutant genes were related to clinical outcomes in EC. Distinct BRCA-associated mutation characteristics were identified in EC and EnOC and associated with diverse PARP inhibitor sensitivities. BRCA-associated features identified two signatures, that were relevant to mutation patterns, clinical outcomes, various levels of immune cell infiltration and expression profiles of immune checkpoint molecules in EC.

## MATERIALS AND METHODS

### Cell culture

KLE and OVCAR4 were cultured in DMEM/F12(1:1) Medium (Boster, Wuhan, China); HEC-1-B were cultured in MEM; A2780, OV1063 and OVCAR8 were cultured in RPMI 1640 (Gibco, Invitrogen, Carlsbad, CA, USA); OV90 and TOV112D were cultured in 1:1 mixture of MCDB 105 medium and medium 199 (Gibco, Invitrogen, Carlsbad, CA, USA), SKOV3 was cultured in McCoy’s 5A (Gibco, Invitrogen, Carlsbad, CA, USA). The mediums were mixed with 10% fetal bovine serum (Gibco, Invitrogen, Carlsbad, CA, USA) and 1% penicillin/streptomycin (Thermo Fisher Scientific). Cells were placed in a 5% CO_2_ and 80% humidity incubator at 37° C.

### Cell viability assay

Cells in the logarithmic growth phase were seeded into a 96-well plate. After 24 hours, the cells were incubated with Olaparib (Selleck, AZD2281, KU0059436) with an initial concentration, and multiple dilutions for 48 hours. Then cell viability was evaluated by cell counting kit 8 (Dojindo, Japan, CK04-500). The absorbance was measured at 450nm by a spectrophotometer (Molecular Devices, SpectraMax ABS Plus), and the cell viability and Olaparib concentration curve were drawn with GraphPad Prism 8.

### Clinical samples and hematoxylin-eosin staining

Tumor samples from patients with EC, EnOC, and En were obtained from Clinical Database and Biobank of Patients with Gynecologic Neoplasms, under ClinicalTrials.gov Identifier NCT01267851 (ethical approval is available at https://clinicaltrials.gov/ct2/show/study/NCT01267851). A pathology review was performed by two pathologists. Informed consent was obtained from all patients. The formalin-fixed and paraffin-embedded specimens were stained with Hematoxylin-Eosin as previously described [26].

### Immunofluorescence

Immunofluorescence was performed as described previously. Cells were transferred on glass overnight, formalin-fixed, permeabilized with triton x-100(9002-93-1, Sigma-Aldrich), incubated with target antibody, and imaged by Olympus fluorescence microscopy (BX53, Olympus). The average fluorescence intensity was analyzed using Image J software.

### Immunohistochemistry

Immunohistochemistry (IHC) was performed as reported previously [26]. We used formalin fixed and paraffin embedded tissue sections to perform IHC staining via Avidin-Biotin Complex Kit (9001, Zsgb-Bio, China) according to the protocols. Positive and negative controls were conducted in each staining. For the evaluation of protein expression in tissues, the staining intensity and the staining percentage were evaluated by Image Pro Plus.

### Transwell migration assay

Briefly, 2 × 10^4^ cells were added to the upper compartment of the Transwell chambers (8μm pore size; Corning Life Sciences). The lower compartment was filled with medium supplemented with 20% FBS, and DMSO or Olaparib. After incubation at 37° C, the upper surface of the filter was washed with PBS and cleared of nonmigratory cells with a cotton swab. The remaining cells at the lower surface of the filter were fixed with paraformaldehyde and stained with 0.1% (wt/vol) crystal violet (Servicebio). Invasive cells were scored by counting the whole filter with a microscope at ×200 magnification.

### TUNEL assay

TUNEL assay was performed according to the protocol of TUNEL Apoptosis Detection Kit (Abbikine, KTA2010). In brief, cells were cultured in microplates and treated with DMSO or Olaparib for 48h. Cells were fixed with 4% paraformaldehyde and permeabilized (0.3% Triton X-100). Then wash the cells with BSA working solution. Prepare reaction mixture and incubate at 37° C for 2h, finally, incubating cells with DAPI. Then take pictures and observe with a fluorescence microscope.

### Flow cytometric analysis

For apoptosis assays, cells were harvested by trypsinization and stained with annexin V-FITC and propidium iodide from an Apoptosis Detection kit (BD 556547) according to the manufacturer’s instructions. For cell cycle analysis, cells were harvested and fixed with 70% ethanol and stained with Propidium Iodide (PI). Data were acquired on a Cytoflex (Beckman Coulter, Pasadena, CA, USA) and analyzed with CytExpert software. The apoptosis rate was identified as the sum of the percentages of early and late apoptosis cells.

### Animal experiments

Female NOD-SCID mice (4 weeks old) were purchased from Beijing HFK Bio-Technology Co. Ltd (Beijing, China). Animal numbers were determined based upon the results of power analysis in combination with previous experience to provide 80% power for a test at a significance level of 0.05. The subcutaneous tumor model of ovarian cancer was established as follows. Briefly, mice were randomly assigned to two groups, four sub-groups. 4.0×10^6^ cells in the mixture of serum-free medium and Matrigel (354230, BD) within 100μL were injected into subcutaneous tissue. After the subcutaneous tumor formation (28 days after injection), the two groups received vehicle or Olaparib (AZD2281, MCE, 50mg/kg/d, intragastric administration) treatment respectively for 21 days. The mice were killed 7 weeks after inoculation with tumor cells and their tumors were excised. Animal experiments were approved by the Animal Ethics Committee of Tongji Hospital. Manipulators were blinded to the group information.

### Sample data acquisition

Integrated genomic and molecular data of 2 777 EC (Tissue selection: Endometrium, Sub-tissue selection: NS, Histology selection: Carcinoma, Sub-histology selection: Endometrioid carcinoma), 423 EnOC (Tissue selection: Ovary, Sub-tissue selection: NS, Histology selection: Carcinoma, Sub-histology selection: Endometrioid carcinoma), and 57 En (Tissue selection: Endometrium, Sub-tissue selection: NS, Histology selection: Hyperplasia, Sub-histology selection: Include all) samples were collected from COSMIC, including a variety of sources, primarily the scientific literature and large international consortia such as CGP, the International Cancer Genome Consortium and The Cancer Genome Atlas (TCGA) [27]. And data was collected until March 2021. Mutation characteristics of EC, EnOC and En were accessible in COSMIC. Sample numbers and corresponding mutations were downloaded for further analysis, such as top mutated genes by tissue, mutation type, mutation counts and mutation frequency. Mutation frequency is the proportion of mutants in patients with a given disease compared to normal wild-type individuals [28].

### Mutation frequency analysis of tumor samples

The EC, EnOC and En samples were downloaded from COSMIC. Then, 5 overlapped mutation genes in EC, EnOC and En were used to analyze the mutation frequency. The top 20 common mutation genes of EC and EnOC were analyzed in COSMIC.

### Molecular characteristics of common mutation genes

Molecular characteristics of 275 common mutation genes were performed in EC and EnOC via cBioPortal with default settings (Uterus- Endometrial Carcinoma-Explore Selected Studies-Cancer Type detailed: Uterine Endometrioid Carcinoma-Query by the 275 genes), including clinical information, cancer type, cancer stage, CNV status, et al. P-value <0.05 was considered statistically significant.

### Co-occurrence mutation genes of BRCA-associated genes

Co-occurrence mutation genes of BRCA-associated genes were performed by cBioPortal (https://www.cbioportal.org/) as previously described [29, 30]. Briefly, mutation genes of samples were performed in EC. Co-occurrence mutation genes and mutual exclusive genes were analyzed by Fisher’s Exact Test.

### Enrichment terms analysis

The enrichment terms analysis was performed to demonstrate the biological processes (GO) and Kyoto Encyclopedia of Genes and Genome (KEGG) pathways of 275 common mutation genes of EC and EnOC in Metascape (http://metascape.org), an online gene annotation tool with multiple authoritative data sources, with default settings [31]. Gene symbols of the 275 genes were imported, then “Homo sapiens” was selected for further analysis to perform the role of the input genes in biological processes and KEGG pathways. The top 20 pathways and process enrichment were showed. The tissue preferences of all the 17791 mutation genes were analyzed in FunRich3.1.3. The signaling pathway enrichment analysis was conducted in cBioPortal with default settings.

### Disease survival analysis

Overall survival, progression free survival, and disease specific survival analysis of EC was performed by cBioPortal (https://www.cbioportal.org/) via Log Rank test. EC samples were clustered into one group with the 275 gene mutation and the other group with the 275 gene wild type. They were also clustered into one group with the genes in Signature 1 mutation, and the other group with the genes in Signature 1 wild type. According to Signature 2, and the five co-occurring mutant genes, EC patients were also clustered into mutation group and wild type group. The Log Rank test gives greater weight to distant differences in outcome events.

### PARP inhibitor sensitivity assessment

The mutation status of EC and EnOC cell lines was obtained from COSMIC. Genomics of Drug Sensitivity in Cancer (https://www.cancerrxgene.org/compounds) were used to assess the sensitivity of these cell lines to PARP inhibitors, including Olaparib, Talazoparib, Rucaparib, and Veliparib. And we also conducted cell counting kit 8 assay to assess PARP inhibitor sensitivity.

### Immune cell infiltration levels evaluation

Samples from COSMIC were grouped with Signature 1 and Signature 2 separately. The expression levels of surface markers of immune cells were analyzed in different groups. Tumor Immune Estimation Resource (TIMER) was a comprehensive resource that could be used to analyze the infiltration of B cell, CD8^+^ T cell, CD4^+^ T cell, macrophage, neutrophil, and dendritic cell across different cancer types systematically [32]. The infiltration levels of CD8^+^ T cells were evaluated here.

### Statistical analyses

SPSS 25.0 was used for statistical analysis. Chi-squared test was used to point mutation frequency. Student’s t-tests and Wilcoxon test were used to evaluate immune cells infiltration level between wild-type group and mutation group. Survival analysis was expressed by the Kaplan-Meier curve, tested by the Log-Rank test. Ordinary one-way ANOVA was used in the comparison between multiple groups. For all analyses, P<0.05 is considered statistically significant unless otherwise specified (Supplementary Table 5).

## Supplementary Material

Supplementary Figures

Supplementary Table 1

Supplementary Table 2

Supplementary Table 3

Supplementary Table 4

Supplementary Table 5
