# Supplementary material for: Genomic mutation features identify distinct BRCA-associated mutation characteristics in endometrioid carcinoma and endometrioid ovarian carcinoma
Source: Aging (Albany NY). 2021 Nov 27;13(22):24686–709. doi: 10.18632/aging.203710 (PMC8660599; doi:10.18632/aging.203710)
Supplement: Supplementary Figures [file aging-13-203710-s001.pdf]

SUPPLEMENTARY FIGURES

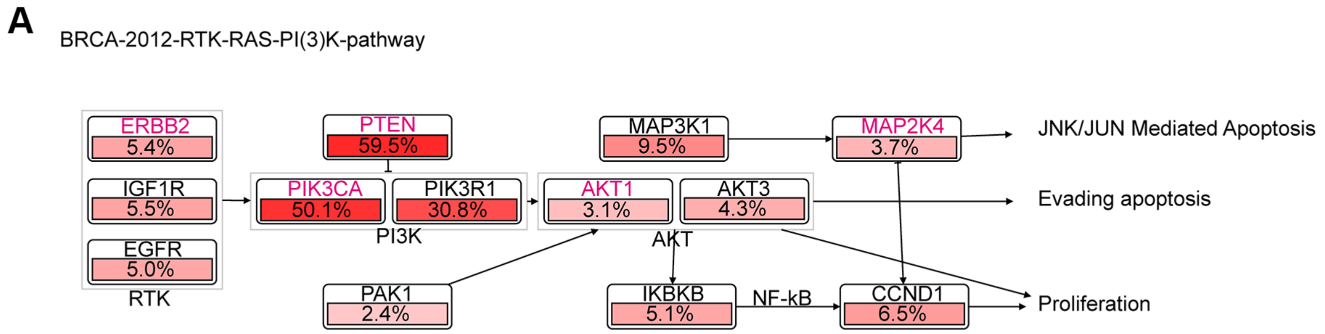

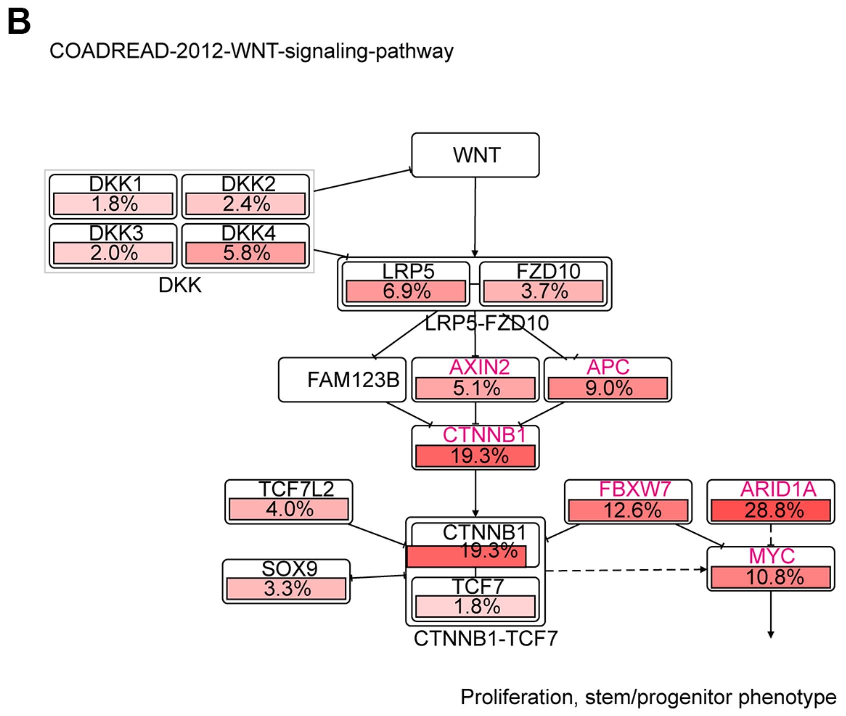

Supplementary Figure 1. Pathway enrichment analysis of 275 common mutation genes in EC. Pathway enrichment analysis of 275 common mutation genes in EC. The genes identified in the pink font were from 275 common mutation genes, percentage indicated the proportion of the gene mutated in EC. (**A**) BRCA-2012-RTK-RAS-PI(3)K-pathway. (**B**) COADREAD-2012-WNT-signaling-pathway.

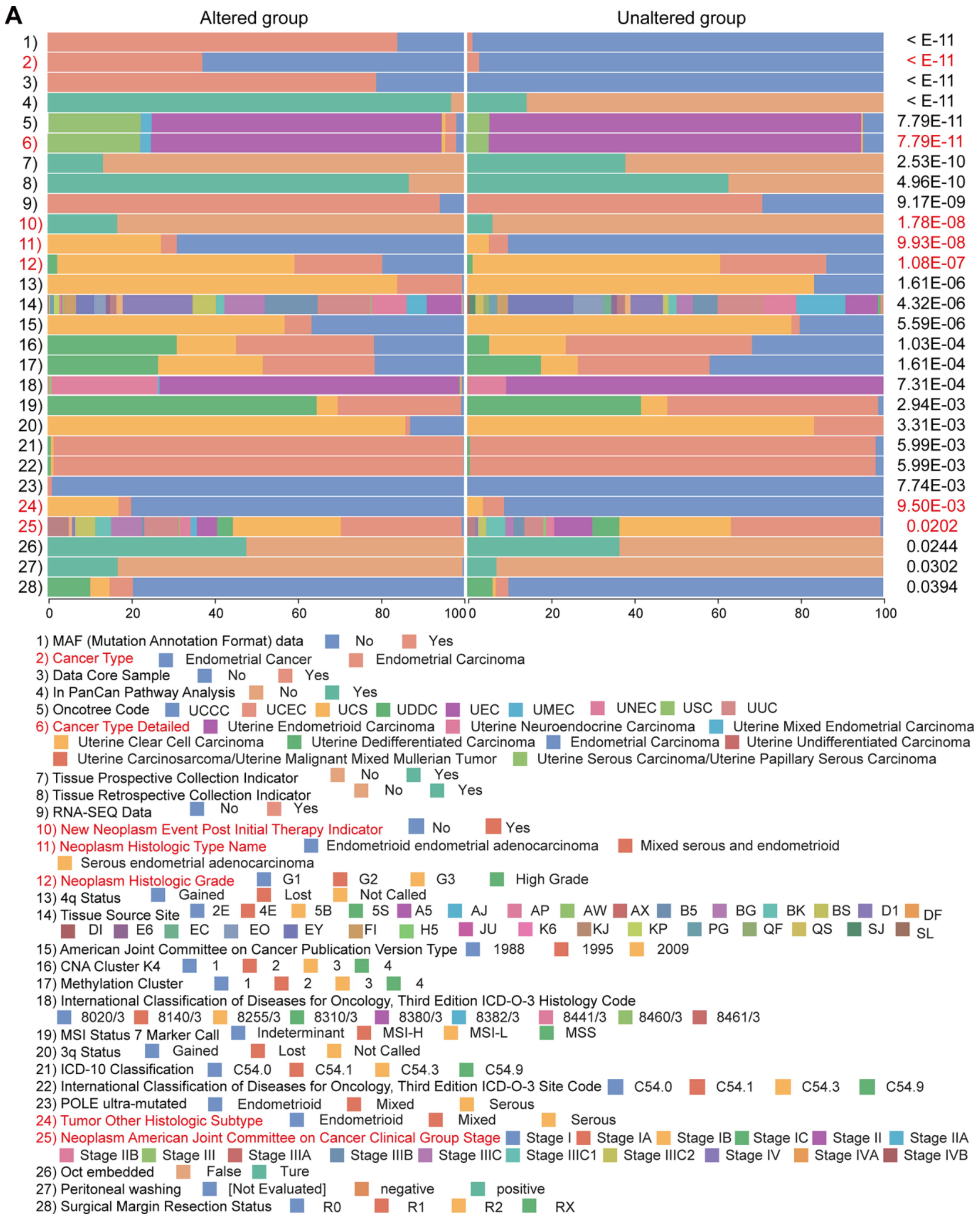

Supplementary Figure 2. Molecular differences in EC patients with/without 275 common mutation genes. (**A**) Molecular differences in EC patients with/without 275 common mutation genes. EC patients with 275 common mutation genes were classified as the altered group, and those without the 275 common mutation genes as the unaltered group.

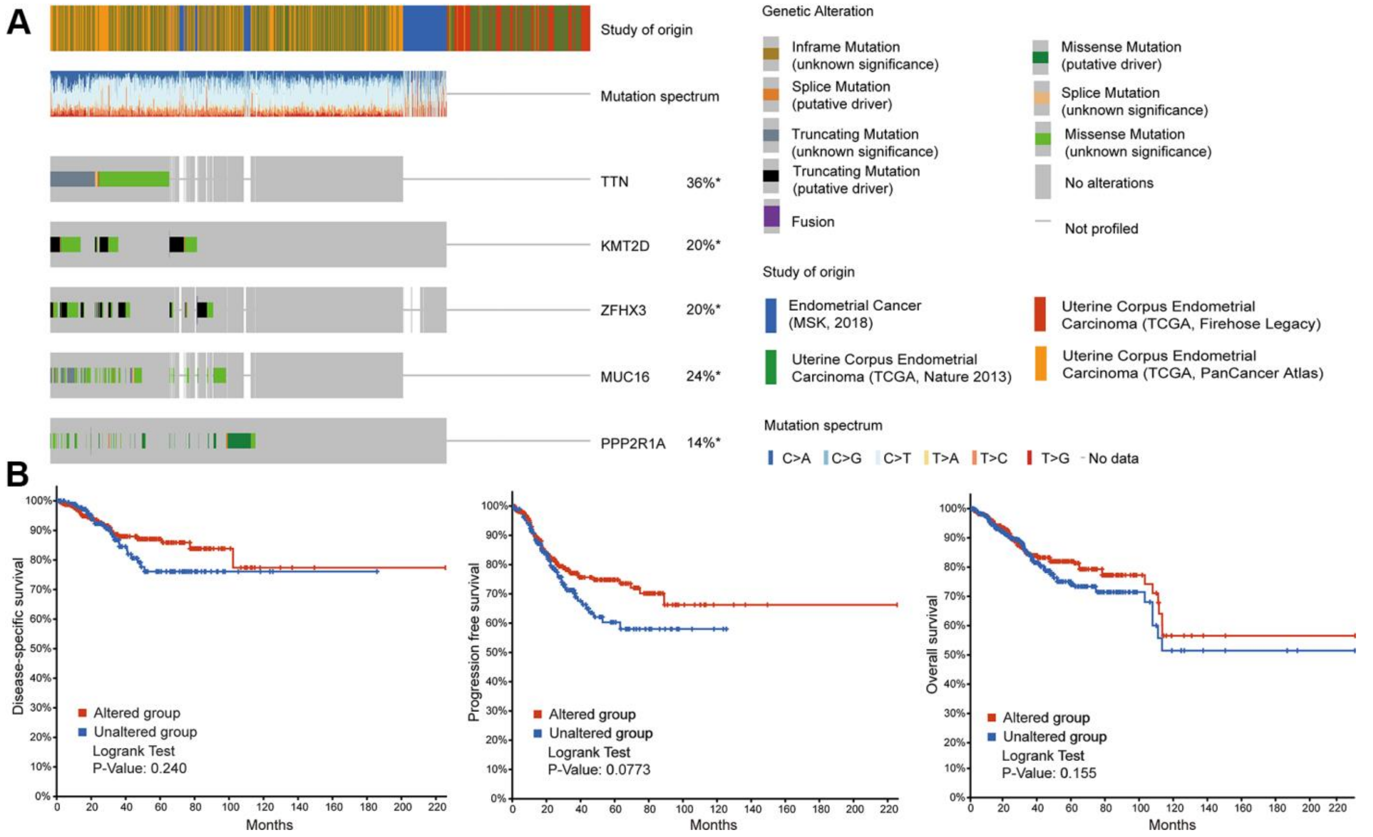

Supplementary Figure 3. Oncoprint a of TTN, KMT2D, ZFHX3, MUC16, PPP2R1A and their association with prognosis in EC patients. (**A**) Oncoprint of TTN, KMT2D, ZFHX3, MUC16, PPP2R1A in EC. (**B**) Kaplan–Meier plots comparing prognostic value (DFS, PFS and OS) in EC patients with/without TTN, KMT2D, ZFHX3, MUC16, PPP2R1A mutation genes. EC patients with TTN, KMT2D, ZFHX3, MUC16, PPP2R1A mutation genes were classified as the altered group, and those without the TTN, KMT2D, ZFHX3, MUC16, PPP2R1A mutation genes as the unaltered group.

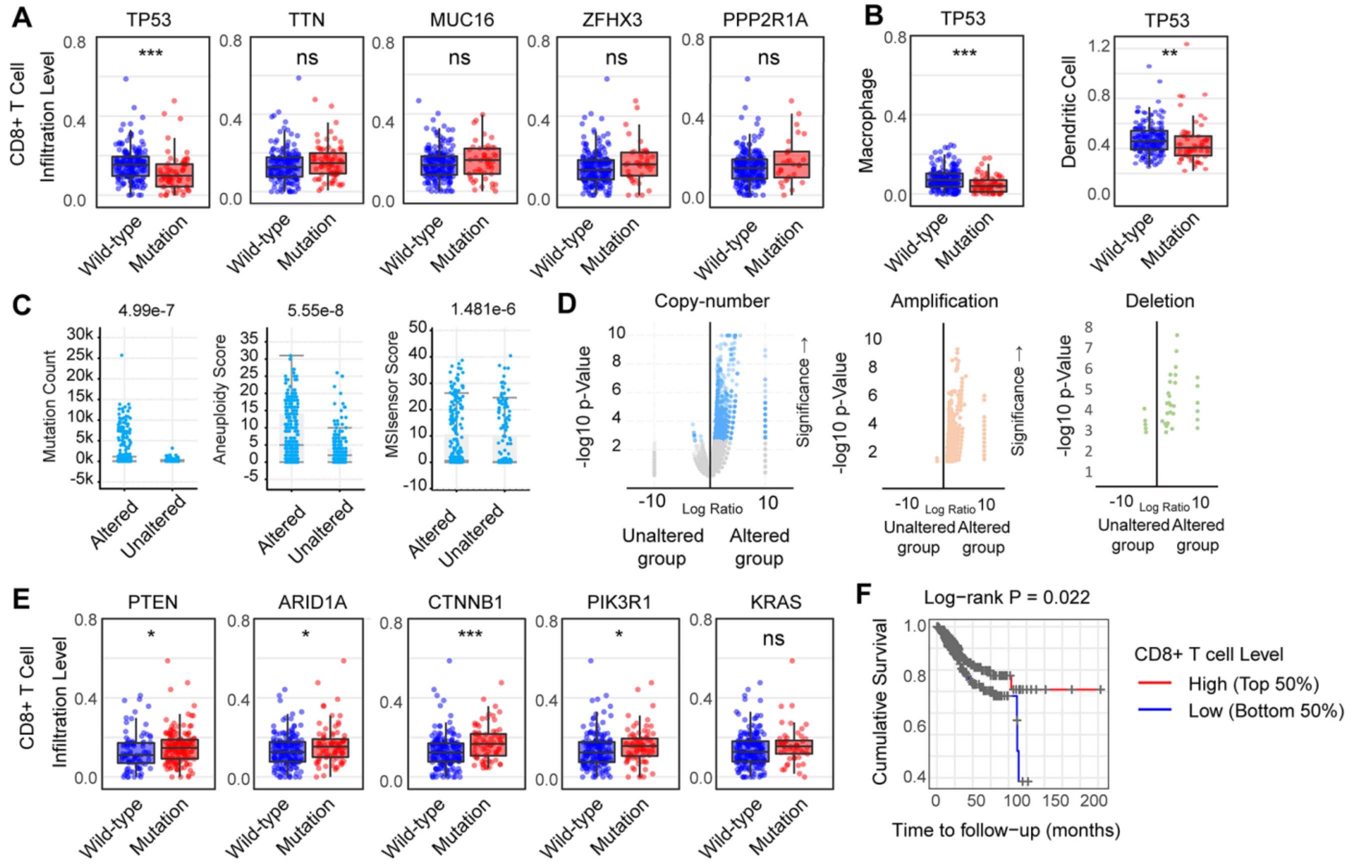

Supplementary Figure 4. Different immune cell infiltrates and genomic characteristics in the two clusters of EC patients with different mutation signatures. (**A**) The infiltration level of CD8+ T cells and mutation status in EC. (**B**) The infiltration level of macrophage and dendritic cells and mutation status of *TP53* in EC (**C**) The mutation count, aneuploidy score, and MSIsensor Score of EC patients clustered by Signature 1. (**D**) The copy-number, amplification, and deletion of EC patients clustered by Signature 1. (**E**) The infiltration level of CD8+ T cells mutation status of *PTEN ARID1A*, *CTNNB1*, *PIK3R1*, and *KRAS* in EC. (**F**) The cumulative survival and CD8+ T cell infiltration level.

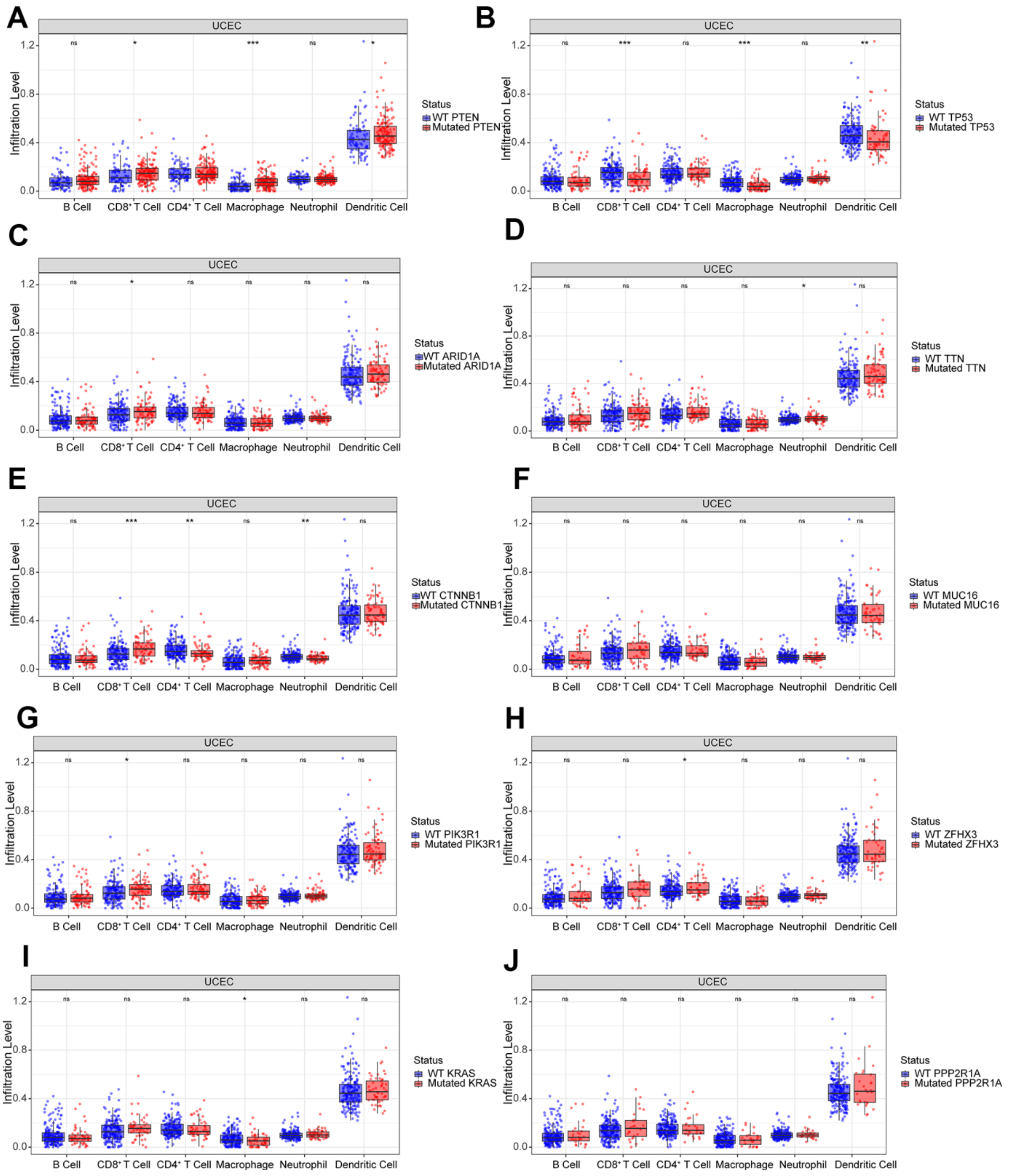

Supplementary Figure 5. Tumor-infiltrating lymphocytes in wild-type or mutation group. (**A**–**J**) The infiltration of B Cell, CD8+ T Cell, CD4+ T Cell, Macrophage, Neutrophil, Dendritic Cell in EC tumors with wild-type or mutation of PTEN, TP53, ARID1A, TTN, CTNNB1, MUC16, PIK3R1, ZFHX3, KRAS and PPP2R1A genes, * P<0.05; ** P<0.01; ***P<0.001; ****P<0.0001, ns indicated that there was no significant statistical difference.

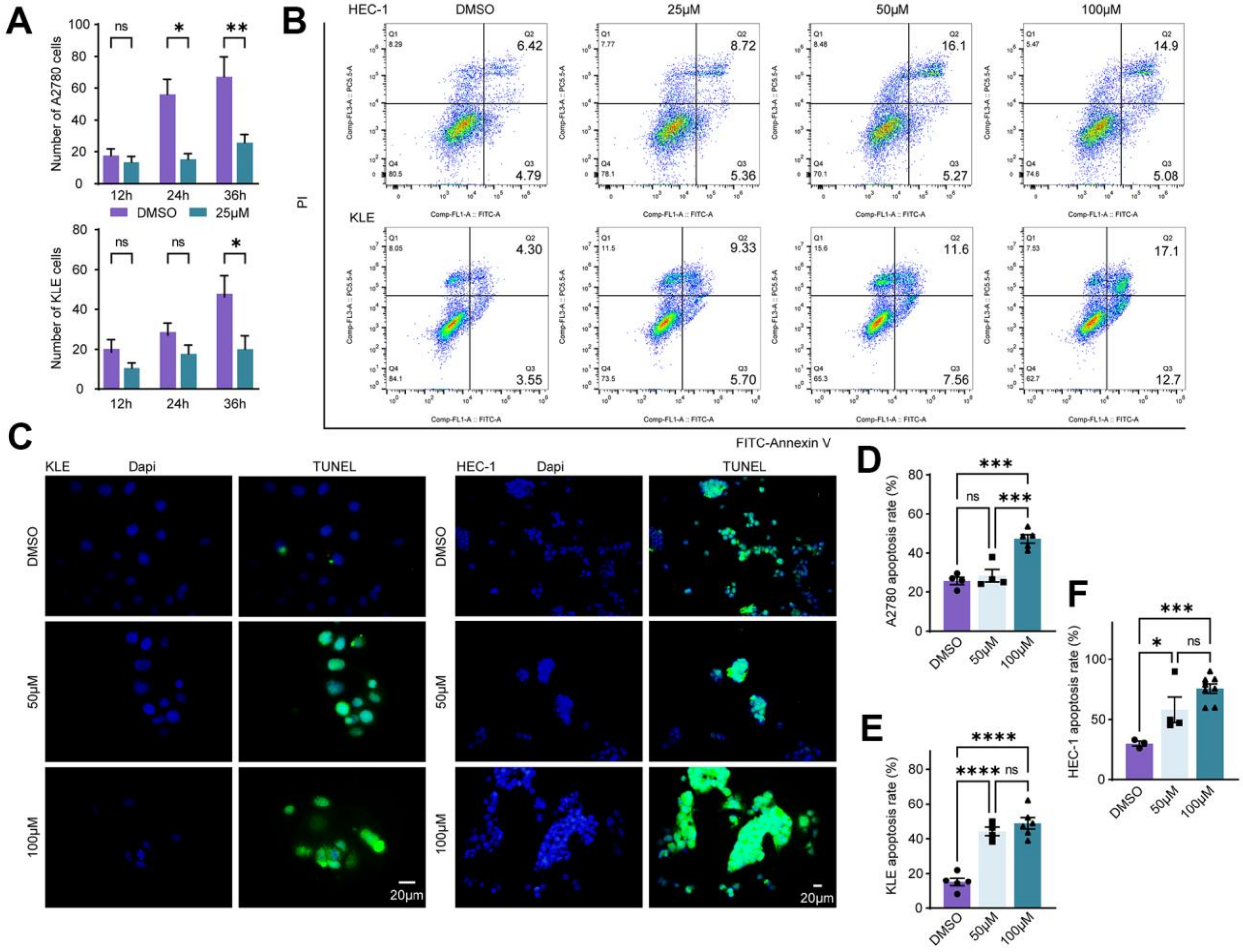

Supplementary Figure 6. Olaparib inhibits cell migration and promotes apoptosis in EC and EnOC *in vitro*. (**A**) The quantification of cell migration assay. (**B**) Apoptosis assay in HEC-1 and KLE. (**C**–**F**) TUNEL assays and their quantifications.

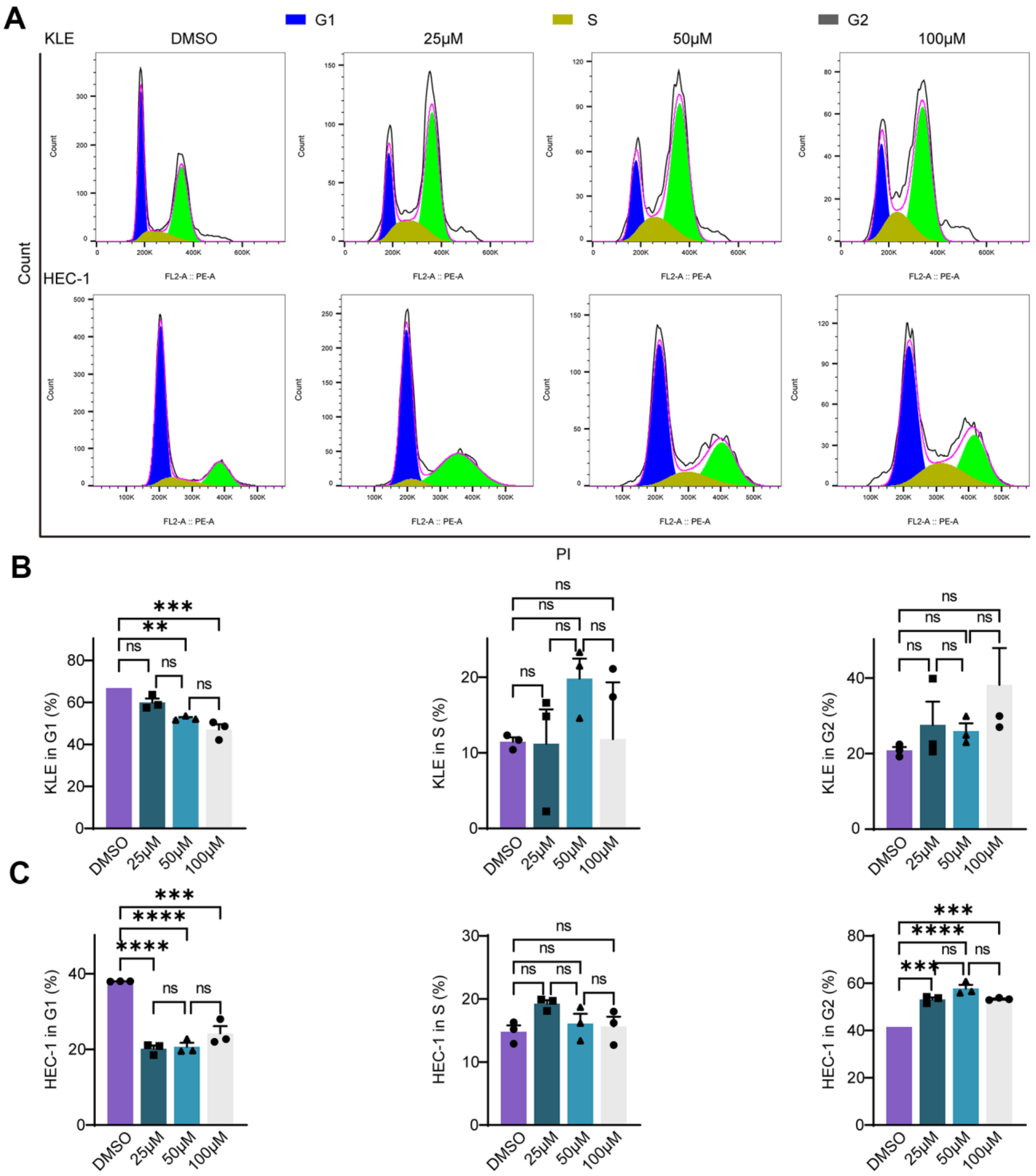

Supplementary Figure 7. Olaparib interferes with the cell cycle in KLE and HEC-1. (**A**–**C**) Cell cycle analysis and their quantifications in KLE and HEC-1.
