# Supplementary material for: Genomic mutation features identify distinct BRCA-associated mutation characteristics in endometrioid carcinoma and endometrioid ovarian carcinoma
Source: Aging (Albany NY). 2021 Nov 27;13(22):24686–709. doi: 10.18632/aging.203710 (PMC8660599; doi:10.18632/aging.203710)
Supplement: Supplementary Table 5 [file aging-13-203710-s006.pdf]

Supplementary Table 5. Clinical attribute and corresponding statistical methods.

Clinical Attribute	Statistical Test
Point mutation frequency	Chi-squared Test
Immune cell infiltration	Student's t-tests and Wilcoxon test
Survival analysis	Log-Rank test
Mutation Count	Kruskal Wallis Test
Aneuploidy Score	Kruskal Wallis Test
MSIsensor Score	Kruskal Wallis Test
MAF (Mutation Annotation Format) data	Chi-squared Test
Cancer Type	Chi-squared Test
Neoplasm Histologic Grade	Chi-squared Test
Data Core Sample	Chi-squared Test
In PanCan Pathway Analysis	Chi-squared Test
Oncotree Code	Chi-squared Test
Cancer Type Detailed	Chi-squared Test
Tissue Prospective Collection Indicator	Chi-squared Test
Tissue Retrospective Collection Indicator	Chi-squared Test
RNA-SEQ Data	Chi-squared Test
New Neoplasm Event Post Initial Therapy Indicator	Chi-squared Test
Neoplasm Histologic Type Name	Neoplasm Histologic Type Name
Neoplasm Histologic Grade	Chi-squared Test
4q Status	Chi-squared Test
Tissue Source Site	Chi-squared Test
American Joint Committee on Cancer Publication Version Type	Chi-squared Test
CNA Cluster K4	Chi-squared Test
Methylation Cluster	Chi-squared Test
International Classification of Diseases for Oncology, Third Edition ICD-O-3 Histology Code	Chi-squared Test
MSI Status 7 Marker Call	Chi-squared Test
3q Status	Chi-squared Test
ICD-10 Classification	Chi-squared Test
International Classification of Diseases for Oncology, Third Edition ICD-O-3 Site Code	Chi-squared Test
POLE ultra-mutated	Chi-squared Test
Tumor Other Histologic Subtype	Chi-squared Test
Neoplasm American Joint Committee on Cancer Clinical Group Stage	Chi-squared Test
Oct embedded	Chi-squared Test
Peritoneal washing	Chi-squared Test
Surgical Margin Resection Status	Chi-squared Test
Apoptosis rate	Ordinary one-way ANOVA
Cell cycle	Ordinary one-way ANOVA
FI	Ordinary one-way ANOVA
Tumor weight	Student's t-test
ki67 (%)	Student's t-test
